# Risk factors for non-closure of an intended temporary defunctioning stoma after emergency resection of left-sided obstructive colon cancer

**DOI:** 10.1007/s00384-020-03559-1

**Published:** 2020-03-24

**Authors:** S. J. van Ommeren–Olijve, J. P. M. Burbach, E. J. B. Furnée, on behalf of the Dutch Snapshot Research Group, H. Algera, G. D. Algie, C. S. Andeweg, F. J. Amelung, T. E. Argillander, M. N. N. J. Arron, K. Arts, T. H. J. Aufenacker, I. S. Bakker, M. van Basten Batenburg, A. J. N. M. Bastiaansen, G. L. Beets, W. A. Bemelman, A. van den Berg, B. van de Beukel, R. L. G. M. Blom, B. Blomberg, E. G. Boerma, F. C. den Boer, F. ter Borg, W. A. A. Borstlap, N. D. Bouvy, J. E. Bouwman, N. D. A. Boye, A. R. M. Brandt-Kerkhof, H. T. Bransma, A. Breijer, W. T. van den Broek, M. E. E Bröker, J. P. M. Burbach, E. R. J. Bruns, T. A. Burghgraef, E. C. J. Consten, R. M. P. H. Crolla, M. Dam, L. Daniels, J. W. T. Dekker, A. Demirkiran, K. W. van Dongen, S. F. Durmaz, A. van Esch, J. A. van Essen, P. Fockens, J. W. Foppen, A. A. W. van Geloven, M. F. Gerhards, E. A. Gorter, W. M. U. van Grevenstein, J. van Groningen, I. A. J. de Groot-van Veen, H. E. Haak, J. W. A. de Haas, P. van Hagen, E. E. van Halsema, J. T. H. Hamminga, K. Havenga, M. van Heinsbergen, B. van den Hengel, E. van der Harst, J. Heemskerk, J. Heeren, B. H. M. Heijnen, L. Heijnen, J. T. Heikens, M. van Heinsbergen, D. A. Hess, N. Heuchemer, C. Hoff, W. Hogendoorn, J. E. van Hooft, A. P. J. Houdijk, N. Hugen, B. Inberg, T. L. Janssen, D. Jean Pierre, W. J. de Jong, A. C. H. M. Jongen, A. V. Kamman, J. M. Klaase, W. Kelder, E. F. Kelling, R. Klicks, G. W. De Klein, F. W. H. Kloppenberg, J. L. M. Konsten, L. J. E. R. Koolen, V. Kornmann, R. T. J. Kortekaas, A. Kreiter, B. Lamme, J. F. Lange, T. Lettinga, D. Lips, G. Lo, F. Logeman, Y. T. van Loon, M. F. Lutke Holzik, C. C. M. Marres, I. Masselink, A. Mearadji, G. Meisen, A. G. Menon, J. W. S. Merkus, D. J. L. M. de Mey, H. C. J. van der Mijle, D. E. Moes, C. J. L. Molenaar, M. J. Nieboer, K. Nielsen, G. A. P. Nieuwenhuijzen, P. A. Neijenhuis, P. Oomen, N. van Oorschot, K. Parry, K. C. M. J. Peeters, T. Paulides, I. Paulusma, F. B. Poelmann, S. W. Polle, P. Poortman, M. H. Raber, R. J. Renger, B. M. M. Reiber, R. Roukema, W. M. J. de Ruijter, M. J. A. M. Russchen, H. J. T. Rutten, J. Scheerhoorn, S. Scheurs, H. Schippers, V. N. E. Schuermans, H. J. Schuijt, J. C. Sierink, P. D. Siersema, C. Sietses, R. Silvis, J. van der Slegt, G. D. Slooter, M. van der Sluis, P. van der Sluis, N. Smakman, D. Smit, A. B. Smits, T. C. van Sprundel, D. J. A. Sonneveld, C. Steur, J. Straatman, M. C. Struijs, H. A. Swank, A. K. Talsma, P. J. Tanis, M. Tenhagen, J. A. M. G. Tol, J. L. Tolenaar, L. Tseng, J. B. Tuynman, M. J. F. van Veen, J. V. Veld, S. C. Veltkamp, A. W. H. van de Ven, L. Verkoele, M. Vermaas, H. P. Versteegh, L. Verslijs, T. Visser, D. van Uden, W. J. Vles, R. J. de Vos tot Nederveen Cappel, H. S. de Vries, S. T. van Vugt, G. Vugts, J. A. Wegdam, T. J. Weijs, B. J. van Wely, M. Westerterp, H. L. van Westreenen, B. Wiering, N. A. T. Wijffels, A. A. Wijkmans, L. H. Wijngaarden, J. H. W. de Wilt, M. van de Wilt, D. D. Wisselink, F. Wit, E. S. van der Zaag, D. D. E. Zimmerman, T. L. R. Zwols

**Affiliations:** 1 Department of Surgery, Isala Clinics, Zwolle, The Netherlands; 2 Department of Surgery, Medical Centre Leeuwarden, Leeuwarden, The Netherlands; 3 Department of Surgery, University Medical Centre Groningen, P.O. Box 30.001, 9700 RB Groningen, The Netherlands

**Keywords:** Colorectal cancer, Surgical oncology, Stoma reversal, Obstructive colon cancer, Non-closure, Defunctioning stoma

## Abstract

**Purpose:**

A substantial part (21–35%) of defunctioning stomas created during resection for colorectal cancer will never be reversed. Known risk factors for non-closure are age, peri- or postoperative complications, comorbidity, and tumor stage. However, studies performed to identify these risk factors mostly focus on rectal cancer and include both preoperative and postoperative factors. This study aims to identify preoperative risk factors for non-reversal of intended temporary stomas created during acute resection of left-sided obstructive colon cancer (LSOCC) with primary anastomosis.

**Methods:**

All patients who underwent emergency resection for LSOCC with primary anastomosis and a defunctioning stoma between 2009 and 2016 were selected from the Dutch ColoRectal Audit, and additional data were collected in the local centers. Multivariable analysis was performed to identify independent preoperative factors for non-closure of the stoma.

**Results:**

A total of 155 patients underwent acute resection for LSOCC with primary anastomosis and a defunctioning stoma. Of these, 51 patients (32.9%) did not have their stoma reversed after a median of 53 (range 7–104) months of follow-up. In multivariable analysis, hemoglobin < 7.5 mmol/L (odds ratio (OR) 4.79, 95% confidence interval (95% CI) 1.60–14.38, *p* = 0.005), estimated glomerular filtration rate (eGFR) ≤ 45 mL/min/1.73 m^2^ (OR 4.64, 95% CI 1.41–15.10, *p* = 0.011), and metastatic disease (OR 6.12, 95% CI 2.35–15.94, *p* < 0.001) revealed to be independent predictors of non-closure.

**Conclusions:**

Anemia, impaired renal function, and metastatic disease at presentation were found to be independent predictors for non-reversal of intended temporary stomas in patients who underwent acute resection for LSOCC. In patients who have an increased risk of non-reversal, the surgeon should consider a Hartmann’s procedure.

## Introduction

Many patients who undergo resection for colorectal cancer, in particular those with severe comorbidities, neo-adjuvant chemoradiation, and/or low anastomosis, receive a defunctioning stoma to reduce the clinical consequences of anastomotic complications. In the emergency setting and especially in the case of malignant bowel obstruction, anastomotic healing might be impaired because of distension of the bowel [1]. For patients with left-sided obstructive colon cancer, the various treatment modalities are available, including colonic stenting, diversion with a stoma only or “blowhole,” resection with end-colostomy, and resection with primary anastomosis with or without diverting stoma.

In the case of resection with primary anastomosis and a diverting stoma, most of the stomas created in these circumstances are intended to be temporary; however, 21–35% of these stomas will never be reversed [2, 3]. Generally, these temporary stomas are loop ileostomies. The morbidity related to loop ileostomies can be considerable, including a high-output stoma, causing dehydration and readmission [4]. Since end colostomies are associated with less serious complications, these stomas may be preferred in the subgroup of patients who have a high risk of non-closure.

Several studies have been published to identify risk factors associated with non-closure of stomas that were intended to be temporary in case of colorectal cancer resection. These risk factors include age, peri- or postoperative complications, comorbidity, and tumor stage [5–8]. However, the majority of these studies concern data on procedures performed for rectal cancer. Less is known about risk factors for non-closure of an intended temporary stoma in the case of (emergency) resection for (left-sided) colon cancer [9]. Moreover, many studies focus on postoperative factors as predictors for non-closure of the stoma, such as anastomotic leakage and other complications [5], whereas few studies focus on preoperative factors, which are the only factors that are useful to estimate the preoperative risk of non-closure of an intended temporary stoma.

This study aims to identify independent preoperative predictors for non-closure of an intended temporary defunctioning stoma that was constructed following emergency resection for a left-sided obstructive colon cancer (LSOCC) with primary anastomosis, in order to aid clinical decision-making and, in addition, to assist informing patients on their individual probability that their stoma could potentially be reversed in the future or not.

## Methods

Recently, a national collaborative retrospective research project has been conducted in the Netherlands by the Dutch Snapshot Research Group (DSRG). The methodology has been described in the first publications of this project [10, 11]. In summary, data from all patients in the Netherlands undergoing resection of primary colorectal cancer are prospectively collected in the Dutch ColoRectal Audit (DCRA). From the latter, the DSRG selected all patients who underwent resection for LSOCC, i.e., all patients with a primary tumor location in the splenic flexure, descending colon, or sigmoid colon, between 2009 and 2016. Patients were considered to have acute colonic obstruction when they had both at least one clinical sign of colonic obstruction (distended abdomen, nausea, and/or vomiting) and radiological signs of colonic obstruction on CT (dilated large and/or small bowel loop). Patients with clinical or radiological signs of perforation were excluded. Of the included patients, short-term data were extracted from the DCRA. In addition, contributors from each participating hospital in the Netherlands were asked to retrospectively provide data from individual patient files on their registered patients with regard to long-term surgical and oncological outcomes. These data were entered into an online tool, following legal privacy regulations. The study was designed and the manuscript prepared in accordance with the STROBE statement [12]. The medical ethics committee of the Academic Medical Centre in Amsterdam reviewed and approved the observational study design, and decided that informed consent was not needed to be obtained as there was no additional burden for the patient owing to the observational design of the study.

### Patient selection

For the current analysis, patients who underwent emergency resection for LSOCC with the construction of a primary anastomosis and defunctioning stoma were selected from the entire cohort. Patients had either a loop ileostomy or a loop colostomy, depending on the surgeon’s preference. If the type of stoma was unknown, the patient was excluded.

Baseline characteristics including age, sex, type of resection and stoma type, and location were collected. Subsequently, patients were subdivided into two groups according to whether the intended defunctioning stoma was reversed at the end of follow-up or not. A set of preoperative factors (known prior to acute resection for LSOCC) hypothesized to be of predictive value for non-closure of the defunctioning stoma was analyzed. These factors included age; body mass index (BMI); classification of the American Society of Anesthesiologists (ASA-classification); preoperative laboratory findings including hemoglobin, leukocyte count, C-reactive protein (CRP), and estimated glomerular filtration rate (eGFR) using the equation developed from the Modification of Diet in Renal Disease (MDRD) study [13]; metastatic disease at presentation; and variables regarding duration of symptoms, such as nausea and vomiting, or absence of stool. Since postoperative factors do not influence intraoperative decision-making, these factors were not included.

### Statistical analysis

For statistical analysis, IBM Statistical Package for the Social Sciences (SPSS) version 23 was used. Values were expressed as means (standard deviation, SD) or medians (range), depending on whether data were normally distributed or not. To identify independent predictors for non-closure of an intended temporary stoma, univariable analysis was performed for every individual preoperative variable as described above by binary logistic regression analysis.

All continuous variables were primarily used as such; however, only when the distribution showed to be highly skewed, we chose to categorize these values for analysis. For hemoglobin, a cutoff value of 7.5 mmol/L was chosen because this value is often used as the lower limit of a normal hemoglobin. For CRP, a cutoff value > 10 mg/L was used as this is the cutoff of CRP in the Glasgow Prognostic Score for predicting cancer outcomes [14]. An eGFR of 45 mL/min/1.73 m^2^ was chosen as cutoff value, as an eGFR < 45 mL/min/1.73 m^2^ implies a clinically relevant decline in renal function.

All variables with a *p* value < 0.2 in univariable analysis were entered together in the multivariable logistic regression model. A manual backward stepwise approach was used to remove non-significant variables; only variables with *p* values < 0.05 were kept in the final multivariable model and considered independent predictors. The odds ratio (OR), 95% confidence interval (CI), and *p* value were reported for every variable identified as independent predictor for non-closure of an intended temporary defunctioning stoma. All reported *p* values are two-tailed, and *p* values < 0.05 were considered statistically significant.

## Results

### Patient characteristics

**Fig. 1:**
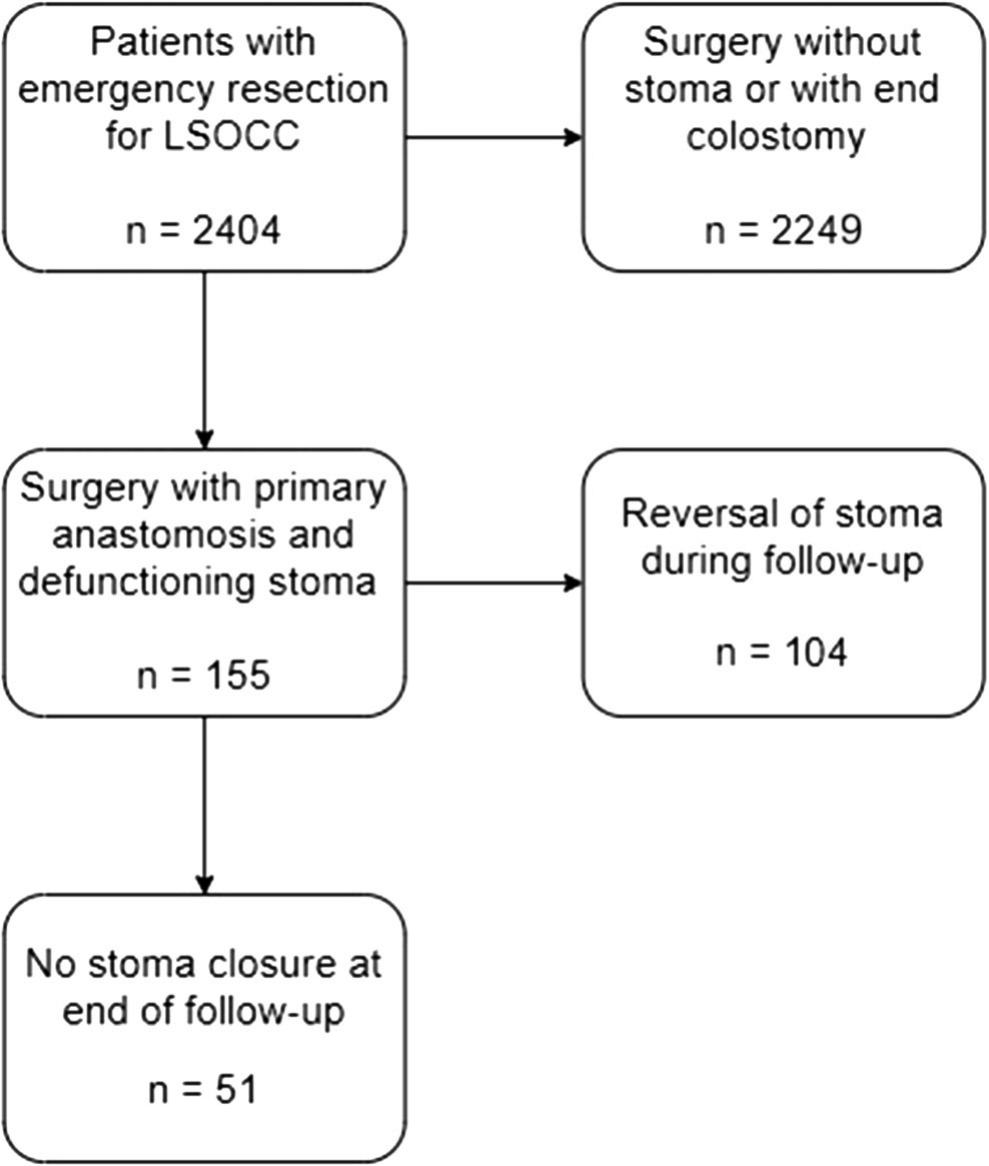
Flow-chart of patient inclusion

A total of 2404 registered patients underwent emergency resection for LSOCC during the study period. Of these patients, 155 patients underwent resection with construction of a primary anastomosis and defunctioning stoma (Fig. 1); a loop ileostomy was constructed in 117 patients (75.5%) and a loop colostomy in 38 patients (25%). The type of resection was a sigmoidectomy in 87 cases (56%), left hemicolectomy in 31 (20%), subtotal colectomy with ileorectal anastomosis in five patients (3%), and partial mesorectal excision (PME) for distal sigmoid colon cancer in 24 patients (16%), and one patient (1%) underwent resection of the transverse colon for a colon cancer at the splenic flexure. The remaining 7 patients (5%) had both a sigmoidectomy and a right hemicolectomy because of an impending blow-out of the caecum. After a median follow-up of 53 (range 7–104) months, the stoma was reversed in 104 patients (67.1%), whereas this was not reversed in 51 patients (32.9%). Thirty-seven of the latter were ileostomies (73%). Baseline characteristics for these two groups of patients are shown in Table 1.

**Table 1:** Baseline characteristics

	Stoma closure *n* = 104	No stoma closure *n* = 51
Male	53 (51.0%)	29 (56.9%)
Female	51 (49.0%)	22 (43.1%)
Age (years)		
−50	3 (2.9%)	5 (9.8%)
50–60	22 (21.2%)	6 (11.8%)
60–70	35 (33.7%)	16 (31.4%)
70–80	34 (32.7%)	13 (25.5%)
80+	10 (9.6%)	11 (21.6%)
BMI (kg/m^2^)	25 (17–38)	24 (18–40)
BMI ≤ 30	80 (85.1%)	39 (83.0%)
BMI > 30	14 (14.9%)	8 (17.0%)
ASA-classification		
ASA 1–2	81 (77.9%)	33 (64.7%)
ASA 3–5	23 (22.1%)	18 (35.3%)
Duration of symptoms prior to presentation (days)	6 (0–173)	5 (0–154)
Preoperative hemoglobin (mmol/L)	8.8 (4.7–11.7)	8.5 (5.2–11.4)
Preoperative leukocyte count (× 10^9^/L)	11.4 (4.1–27.2)	11.1 (4.7–24.0)
Preoperative C-reactive protein (mg/L)	10 (1–239)	22 (1–290)
Preoperative eGFR (mL/min/1.73 m^2^)	83 (32–154)	76 (12–130)
Clinical M-stage		
cM0	92 (91.1%)	32 (64.0%)
cM1	9 (8.9%)	18 (36.0%)
Type of resection		
Sigmoidectomy	61 (58.7%)	26 (51.0%)
Left hemicolectomy	20 (19.2%)	11 (21.6%)
Subtotal colectomy	2 (1.9%)	3 (5.9%)
Low anterior resection (partial mesorectal excision)	18 (17.3%)	6 (11.8%)
Sigmoidectomy + right hemicolectomy	3 (2.9%)	4 (7.8%)
Transverse colon resection	0 (0.0%)	1 (2.0%)
Approach of operation		
Open	92 (88.5%)	51 (100.0%)
Laparoscopic	12 (11.5%)	0 (0.0%)
Stoma location		
Ileum	80 (76.9%)	37 (72.5%)
Ascending colon	1 (1.0%)	3 (5.9%)
Transverse colon	21 (20.2%)	7 (13.7%)
Descending colon	2 (1.9%)	4 (7.8%)
Pathological tumor stage		
pT2	3 (2.9%)	0 (0.0%)
pT3	74 (71.2%)	34 (66.7%)
pT4	27 (26.0%)	17 (33.3%)
pN0	48 (46.2%)	17 (33.3%)
pN1	34 (32.7%)	19 (37.3%)
pN2	22 (21.2%)	15 (29.4%)

Data are given as number of cases (%). Continuous variables were given as median (range)

*BMI*, body mass index; *ASA*, American Society of Anesthesiologists; *eGFR*, estimated glomerular filtration rate

During presentation, abdominal distension was present in 130 patients (83.9%) and vomiting in 94 (61%), and 22 patients (14%) reported weight loss. In 122 cases (78.7%), abdominal CT-scan was performed prior to surgery, which showed distended bowels in 76 patients (64%). Imaging showed distant metastasis in 27 patients (17%) at presentation.

### Predictors for non-closure of stoma

In univariable analysis, hemoglobin < 7.5 mmol/L, CRP > 10 mg/L, eGFR ≤ 45 mL/min/1.73 m^2^, and metastatic disease at presentation showed to be significantly correlated with an increased risk of non-closure of the intended temporary stoma (Table 2). Age; type of stoma, i.e., ileo- or colostomy; BMI; ASA-classification; and duration of symptoms were not identified as significant predictors in univariable analysis.

**Table 2:** Univariable and multivariable analysis

	Univariable analysis OR (95% CI)	*P* value	Multivariable analysis OR (95% CI)	*P* value
Age (years)		0.576		
≤ 70	1.00 (reference)			
> 70	1.21 (0.62–2.38)			
Sex		0.490		
Male	1.00 (reference)			
Female	0.79 (0.40–1.55)			
BMI (kg/m^2^)	0.99 (0.91–1.07)	0.783		
ASA-classification		0.083		
ASA 1–2	1.00 (reference)			
ASA 3–5	1.92 (0.92–4.02)			
Duration of symptoms prior to presentation (days)	1.01 (0.99–1.02)^a^	0.319		
Preoperative hemoglobin		*0.027*		*0.005*
> 7.5 mmol/L	1.00 (reference)		1.00 (reference)	
< 7.5 mmol/L	2.96 (1.14–7.72)		4.79 (1.60–14.38)	
Preoperative leukocyte count (× 10^9^/L)	0.99 (0.91–1.07)	0.736		
Preoperative C-reactive protein		*0.014*	**NS**	
≤ 10 mg/L	1.00 (reference)			
> 10 mg/L	2.53 (1.28–5.31)			
Preoperative eGFR		*0.022*		*0.011*
> 45 mL/min/1.73 m^2^	1.00 (reference)		1.00 (reference)	
≤ 45 mL/min/1.73 m^2^	3.62 (1.21–10.84)		4.64 (1.41–15.10)	
Clinical M-stage		*< 0.001*		*< 0.001*
cM0	1.00 (reference)		1.00 (reference)	
cM1	5.75 (2.35–14.08)		6.12 (2.35–15.94)	
Type of stoma		0.597		
Ileostoma	1.00 (reference)			
Colostoma	1.23 (0.57–2.65)			

*OR (95% CI)*, odds ratio (95% confidence interval); *BMI*, body mass index; *eGFR*, estimated glomerular filtration rate; *NS*, not significant; *ASA*, American Society of Anesthesiologists

The significant *p*-values in uni- and multivariable analysis are highlighted in italic

All variables with *p* values < 0.2 in univariable analysis, i.e., ASA-classification, hemoglobin < 7.5 mmol/L, CRP > 10 mg/L, eGFR ≤ 45 mL/min/1.73 m^2^, and metastatic disease at presentation, were entered into the multivariable logistic regression analysis. Only hemoglobin < 7.5 mmol/L (OR 4.79, 95% CI 1.60–14.38, *p* = 0.005), eGFR ≤ 45 mL/min/1.73 m^2^ (OR 4.64, 95% CI 1.41–15.10, *p* = 0.011), and metastatic disease (OR 6.12, 95% CI 2.35–15.94, *p* < 0.001) were found to be independent predictors of non-closure of defunctioning stoma (Table 2).

## Discussion

Patients with anemia, impaired renal function and/or metastatic disease at presentation had a significantly higher risk that their intended temporary defunctioning stoma was not reversed after emergency resection for LSOCC with primary anastomosis. In contrast, the type of temporary stoma, i.e., ileo- or colostomy, BMI, sex, age, or ASA-score, did not affect the rate of stoma reversal.

This study revealed a high non-closure rate of defunctioning stomas as approximately one-third of defunctioning stomas was not reversed. However, this is comparable with the percentages reported in the literature [3]. To the best of our knowledge, this is the first study identifying risk factors of non-closure of an intended temporary stoma in case of LSOCC, and, in addition, the first study only focusing on preoperative parameters. The results of the current study can contribute to clinical decision-making in patients who undergo an emergency left-sided colon resection for obstruction. When one or even more of the identified risk factors for non-closure are present, the operating surgeon should consider creating an end-colostomy instead of a primary anastomosis with loop ileostomy, as the latter has a higher risk of high output and consequently dehydration which often leads to readmission [4]. The preoperative presence of these risk factors can assist in counseling patients about the potential risk of non-closure of a temporary stoma and in shared decision-making with regard to the choice of stoma type. In addition, one can also consider the prevention of an emergency resection. Initial decompression might prevent postoperative mortality and non-closure of a stoma, especially in the elderly patients [2, 15]. Such a bridging strategy can be accomplished by using a colonic stent or just a defunctioning stoma. Resection can be scheduled in an elective setting after the bowel distention has been restored.

A low hemoglobin level and impaired renal function are both expressions of a poor physical condition and/or a longer course of the disease. This might explain a four times higher risk of non-closure of a stoma in patients with one of these risk factors. Duration of symptoms prior to presentation also provides information about the length of disease. Therefore, we also expected this variable to be significantly associated with a lower stoma reversal rate. However, duration of symptoms was not identified as an independent predictor of non-closure of the stoma in the current study. The reason for this might be the unreliably reporting of this factor as a result of the retrospective design of the study. In addition, symptoms usually develop gradually and duration of symptoms is a subjective measurement obtained from patients in an acute phase of their disease. Metastatic disease at presentation was associated with a six times higher risk of non-closure of the stoma. This could at least partly be explained by a shorter survival of patients with stage IV disease. Furthermore, patients with metastatic disease often undergo systemic therapy that interferes with surgical interventions, patients might be less motivated to have their stoma reversed due to the associated risks of surgery during systemic therapy, and surgeons might be less willing to reverse the stoma in the palliative setting. In addition, one should avoid an ileostomy and consider performing a Hartmann’s procedure in the case of metastatic disease, as chemotherapy could lead to high stoma output in patients with an ileostomy.

Other factors that are normally taken into account when choosing between a defunctioning stoma and end-colostomy, such as age and ASA-score, were not identified as independent predictors of non-closure of the stoma in the current study. Therefore, it seems not justifiable to base one’s decision on these factors, despite the fact that previous studies have shown age to be a predictive factor for non-reversal of a defunctioning stoma in rectal cancer surgery [9]. In addition, BMI was also not found to be associated with non-closure of the stoma in this study, despite our clinical impression that patients with obesity generally have a more complicated postoperative course and therefore are less likely to have their stoma reversed. This might be well explained by the relatively low number of patients with obesity in this cohort.

Previous studies reported complications from index surgery to be significantly associated with non-closure of stoma [8, 9]. However, this is only known after the index surgery, whereas the decision of creating a stoma is taken before or during surgery. We therefore only focused on preoperative variables in the current study. Moreover, previous studies mainly focused on surgery for rectal cancer; none of these studies concerns colon cancer.

The current study has some limitations. First, the study design was retrospective, incorporating the risk of bias as well as some missing data, although the latter was limited for the included study population; for hemoglobin, 3.2% of the data was missing, for eGFR 4.5% and for CRP 7.1%. In 2.6% of patients, it was unknown whether they had metastasis at presentation or not. Although a prospective study design would be preferable, the current study design enabled us to collect a relatively large cohort of patients from a representative variety of hospitals, which is important as emergency left-sided colon resection with primary anastomosis and a defunctioning stoma is relatively rare. In addition, oncological follow-up regarding local recurrence or metachronic metastasis has not been taken into account in this analysis, whereas this might be an important factor in the (postoperative) decision whether to reverse a stoma or not. Lastly, intraoperative findings such as peritonitis, bowel perfusion, or advanced tumor stage could strengthen or weaken the model that estimates the risk of non-reversal in the current study. However, due to the retrospective design, these intraoperative data were not available for analysis in the current study.

In conclusion, hemoglobin < 7.5 mmol/L, eGFR ≤ 45 mL/min/1.73 m^2^, and metastatic disease at presentation were found to be independent preoperative predictors for non-closure of an intended temporary defunctioning stoma created during acute resection for left-sided obstructive colon cancer with primary anastomosis. These findings might support clinical decision-making on the type of stoma creation and might assist informing patients on their individual probability that their stoma could potentially be reversed in the future or not.

## Data Availability

Data available on request, availability of material not applicable
